# Supplementary figures and images for: Identification of hypertension gene expression biomarkers based on the DeepGCFS algorithm
Source: PLoS One. 2025 Jan 24;20(1):e0314319. doi: 10.1371/journal.pone.0314319 (PMC11761172; doi:10.1371/journal.pone.0314319)

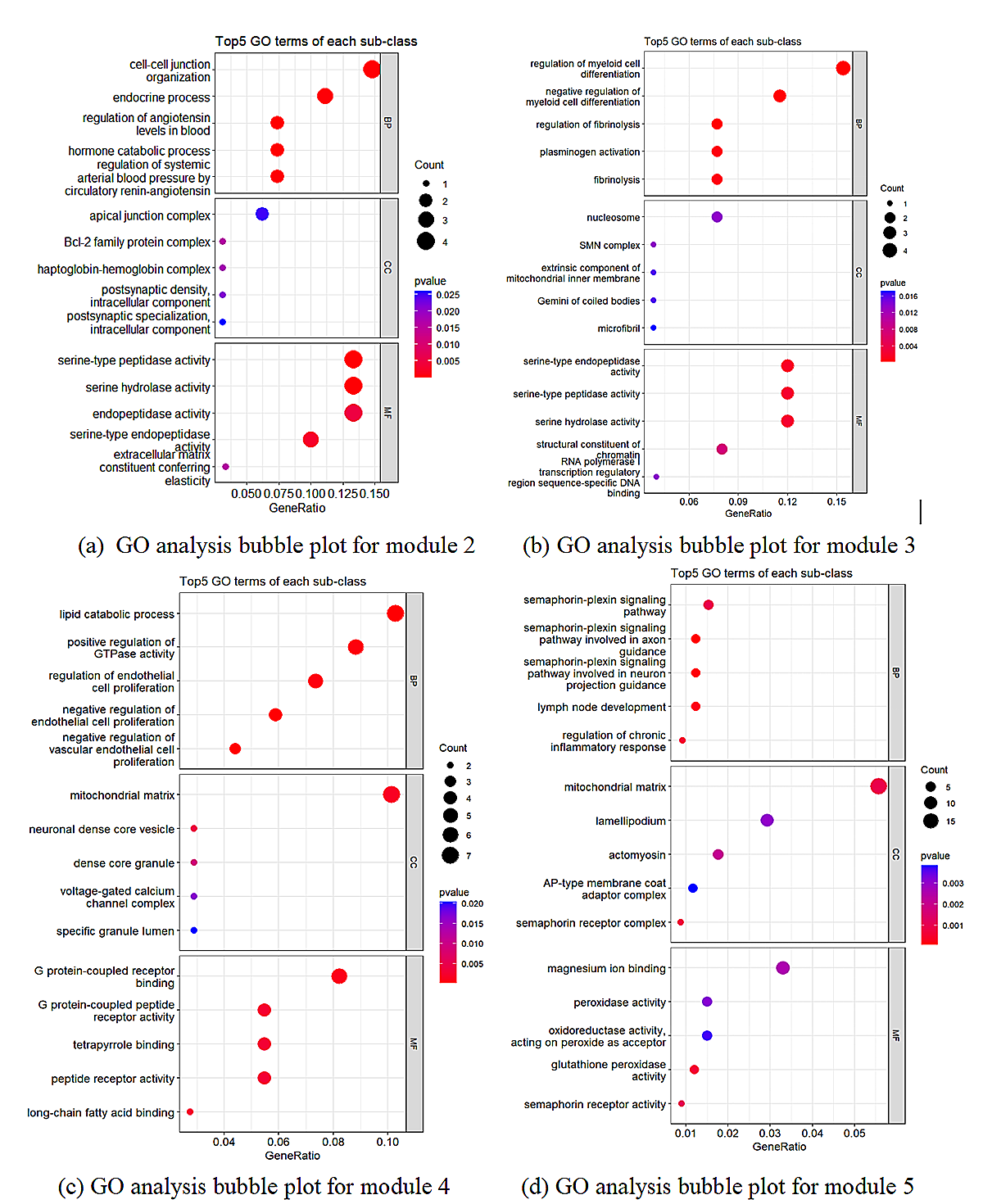

Supplement: S1 Fig — (TIF) [file pone.0314319.s001.tif]

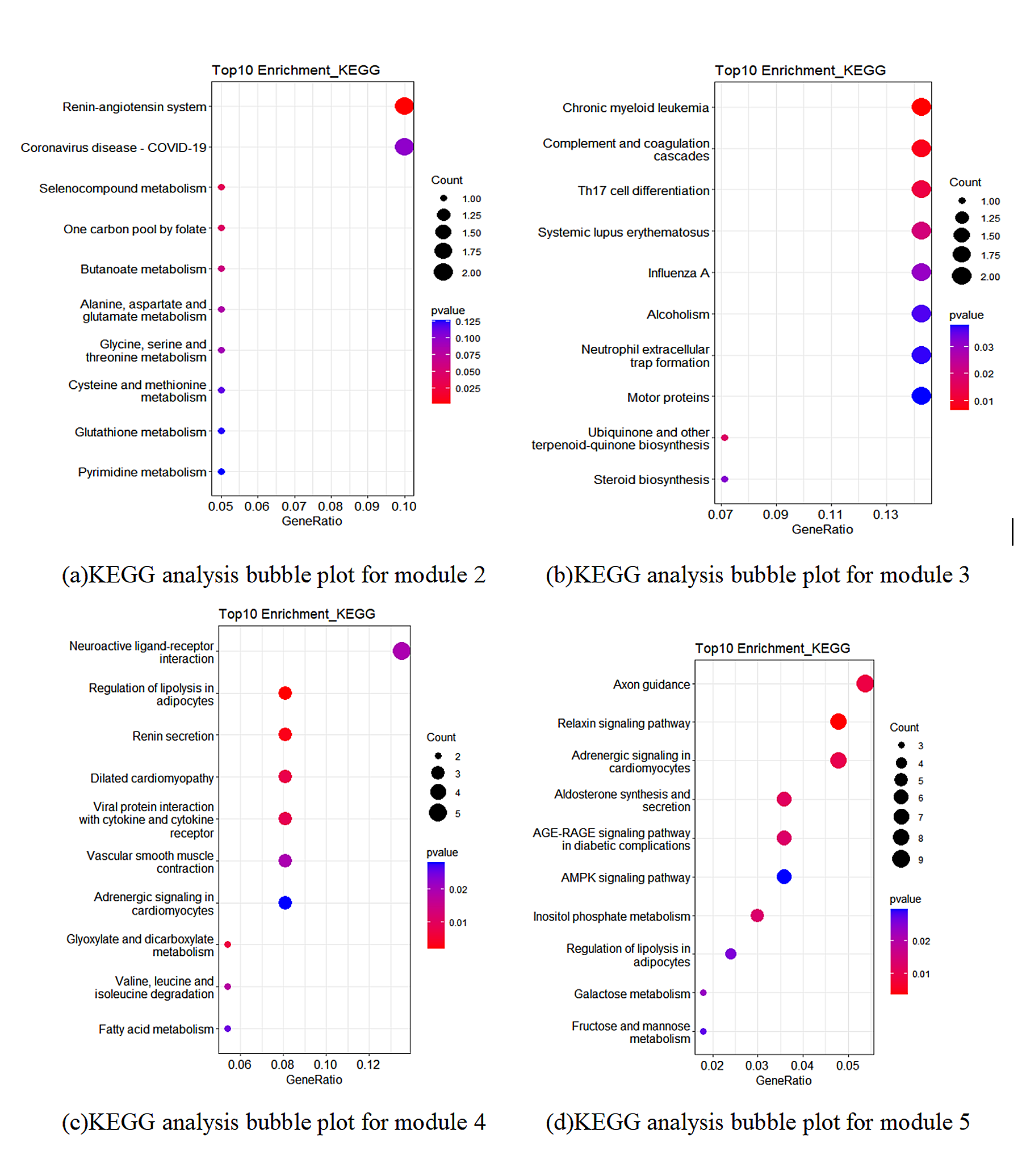

Supplement: S2 Fig — (TIF) [file pone.0314319.s002.tif]

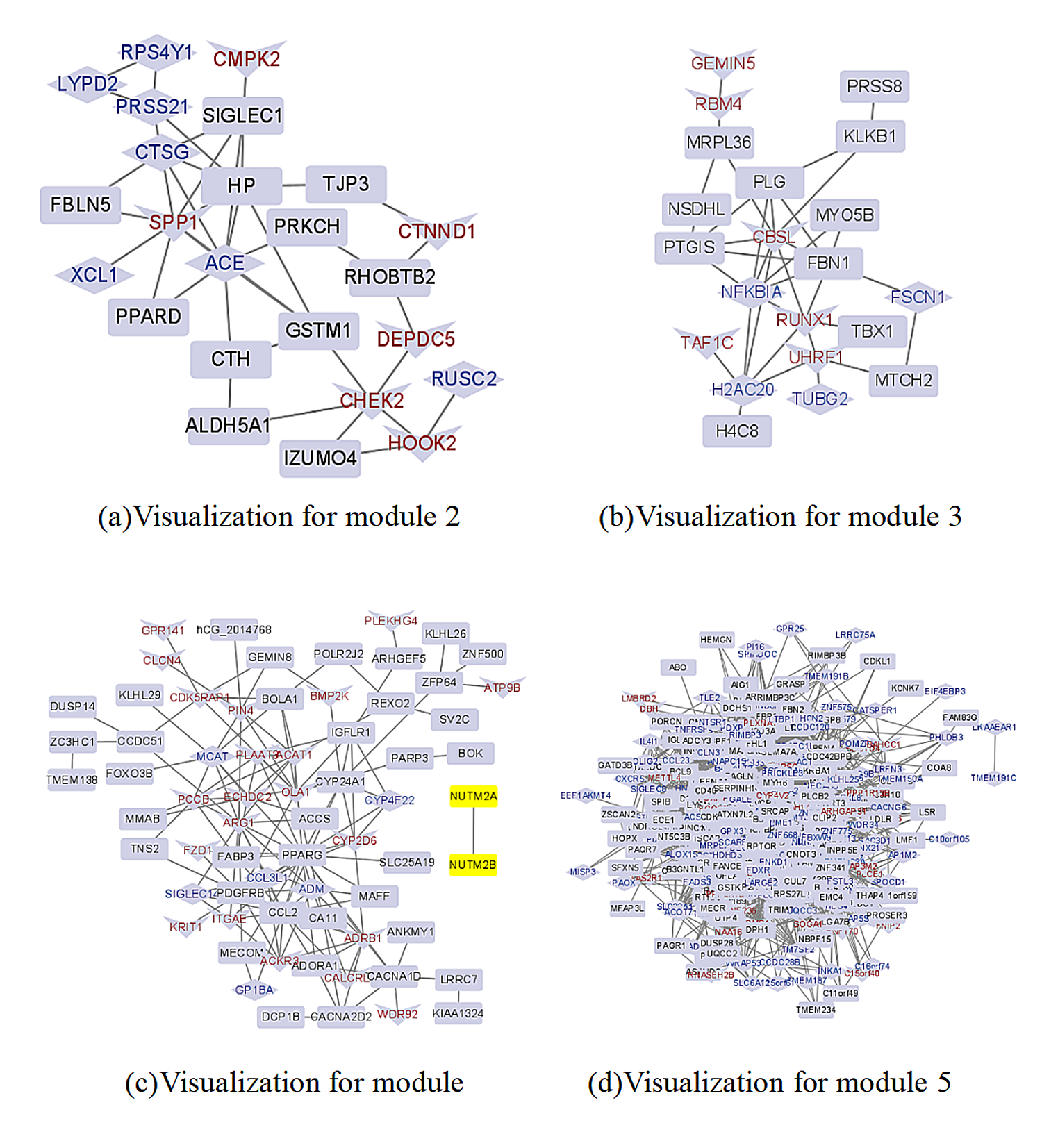

Supplement: S3 Fig — (TIF) [file pone.0314319.s003.tif]

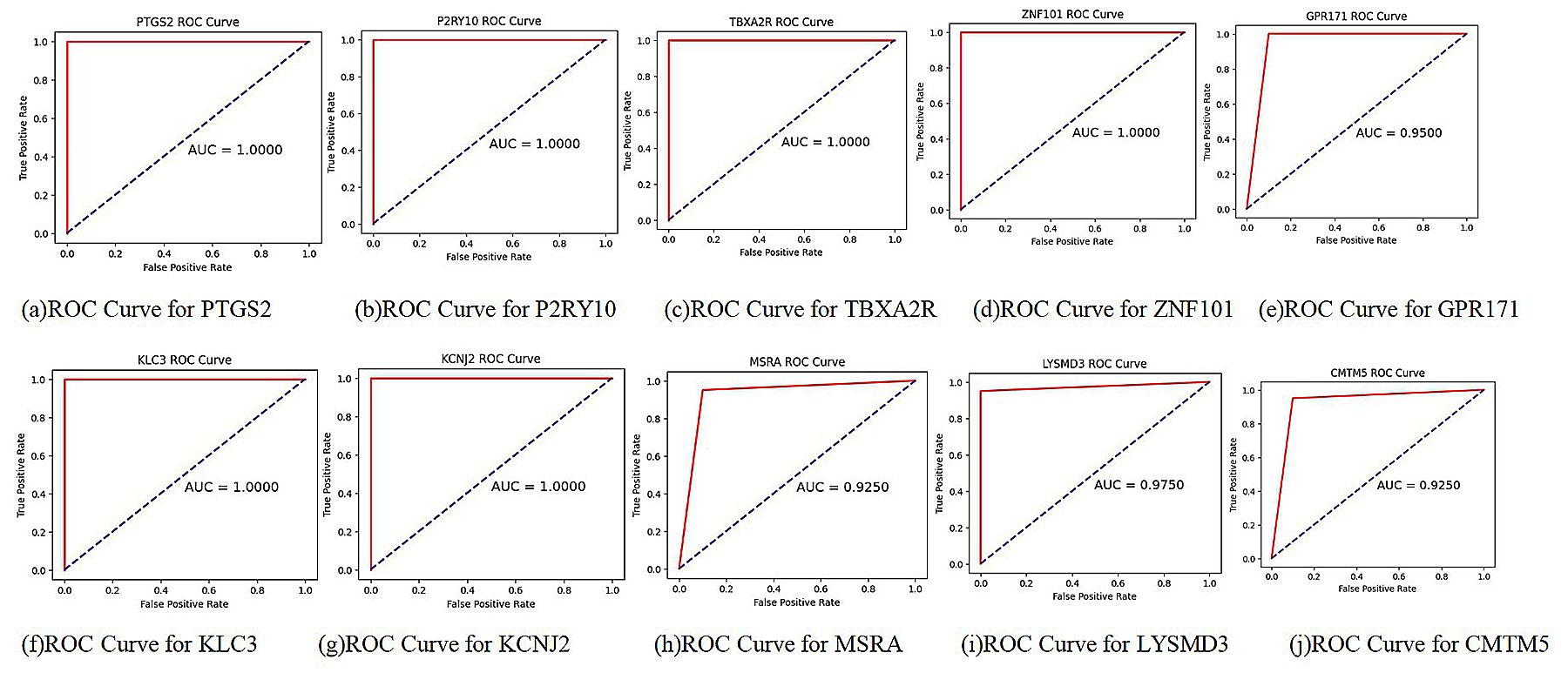

Supplement: S4 Fig — (TIF) [file pone.0314319.s004.tif]

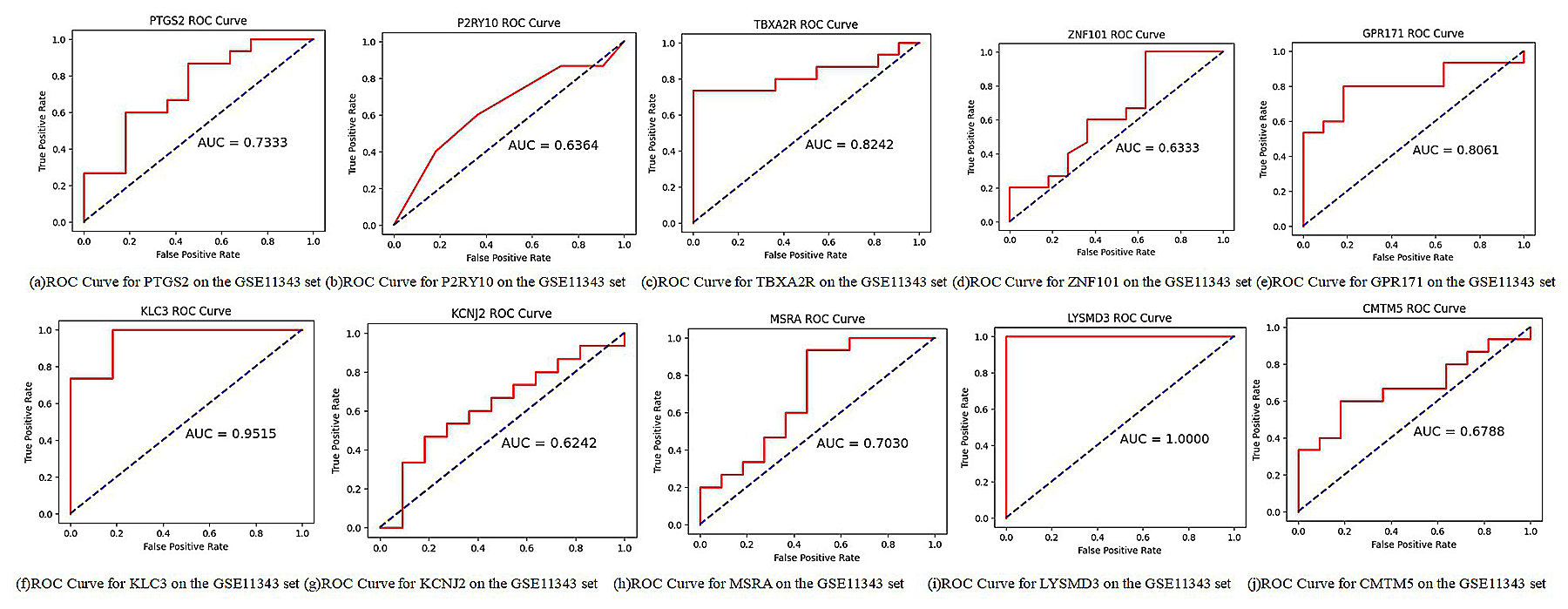

Supplement: S5 Fig — (TIF) [file pone.0314319.s005.tif]
