# Supplementary material for: Identification of hypertension gene expression biomarkers based on the DeepGCFS algorithm
Source: PLoS One. 2025 Jan 24;20(1):e0314319. doi: 10.1371/journal.pone.0314319 (PMC11761172; doi:10.1371/journal.pone.0314319)
Supplement: Attachment — Submitted filename: Response to Reviewers.pdf [file pone.0314319.s006.pdf]

Response Letter

(Manuscript ID: PONE-D-23-39381)

Dear Babatunde OlanrewajuMotayo, Ph.D. and Reviewers,

Thanks very much for taking your time to review our manuscript (Titile: Identification of Hypertension Gene Expression Biomarkers Based on the DeepGCFS Algorithm). We really appreciate all your comments and suggestions! In this revision, we have addressed all of these comments. We hope the revised manuscript has now met the publication standard of ***PLOS ONE***.

On the next pages, our point-to-point responses to the comments raised by the reviewers and the additional editor are listed.

Thanks again!

Yours Sincerely,

Zongjin Li
E-mail: 202033341012@stu.qhnu.edu.cn

SUGGESTIONS FROM EDITOR

Academic Editor

Comment: Thank you for submitting your manuscript to PLOS ONE. After careful consideration, we feel that it has merit but does not fully meet PLOS ONE's publication criteria as it currently stands. Therefore, we invite you to submit a revised version of the manuscript that addresses the points raised during the review process.
Response: Thank you for the comments and suggestions concerning our manuscript. They are valuable and very helpful for revising and improving our manuscript and have important guiding significance to our research. We have studied the comments carefully and made corrections. We have revised the manuscript style as required by the journal.

SUGGESTIONS FROM REVIEWERS

REVIEWER #1

Comment #1: In the introduction, discuss the advantages and disadvantages of the reviewed literature methods for identifying hypertension biomarkers.
Response #1: Thank you for the reviewers' suggestions. In the introduction section, we have included the advantages and disadvantages of methods used for identifying hypertension biomarkers in the literature review. The specific changes are as follows:

...Gao et al.[3] when exploring potential biomarker genes associated with hypertension, firstly, carried out DEG through the limma package, then obtained the interactions network between genes through the STRING platform, subsequently, applied three centrality algorithms (degree centrality, median centrality, and subgraph centrality) in the CytoNCA plugin to identify the hub genes in the network, and finally revealed the critical roles of Cyp4b1, Cyp4a31, Loxl2, and TFs, Esr1, Pparg, and Rxrg in the development of hypertension. However, DEG, by setting specific thresholds, may overlook hub genes. Indeed, many genes involved in the pathologic process of hypertension may be minimally expressed but critically important, and these genes are likely to be excluded under strict threshold criteria. Furthermore, this method often overlooks structural and attribute information among genes, which are crucial for unraveling the pathogenesis of hypertension and identifying functionally critical genes.

... Li et al. [5] utilized WGCNA to construct co-expression networks of mRNA and miRNA in hypertension, uncovering two key gene modules and one key miRNA module. By calculating indicators such as module significance, module membership, and network centrality, they identified 12 gene markers and ten miRNA markers for hypertension. However, this method overlooks potential local expression relationships that may exist only among specific genes and inadequately handles higher-order associations, potentially missing some deeper connections within complex biological systems.

... Reel et al.[8] in exploring biomarkers distinguishing different types of hypertension, they employed random forest and correlation-based feature selection

methods to filter multidimensional omics data features, and they utilized machine learning classifiers to study the classification of five different types of hypertension diseases. Ultimately, they identified has-miR-15a-5p and two plasma metabolites (C9 and PC ae C38:1) as biomarkers. Although this study exhibits advantages in integrating multi-omics data through machine learning methods, it solely relies on machine learning methods for biomarker identification. It fails to consider the topological structure among features fully. Jiang et al.[9] in their study of genetic diagnostic biomarkers for pulmonary arterial hypertension, they first screened out the differential genes by DEG, then used LASSO regression and SVM, two feature selection methods, to identify biomarkers. Eventually, they selected CALD1 and SLC7A11, jointly identified by the two methods, as biomarkers. Although the feature selection methods perform well in identifying hypertension biomarkers, there are some limitations...

Comment #2: The phrase "They also do not take into account the attribute information of features" means that existing methods may overlook the specific characteristics or attributes of features when identifying biomarkers. This needs more clarification on how these attributes are important.

Response #2： Thanks for this comment from the reviewer. In the introduction section, we have included the importance of feature attributes.

Comment #3: Expand on the sentence about feature selection methods, detailing various types such as wrapper and filter selections. Highlight that while wrapper methods capture some feature relationships through model performance, they may not explicitly model dependencies among features, which is crucial in biomarker identification.

Response #3： Thank you for the reviewers' suggestions. In the introduction section, we have expanded upon the description of feature selection methods. The specific changes are as follows:

...filtered feature selection is computationally fast and easy to understand but may ignore complex interactions between features. Wrapper feature selection, although capable of capturing indirect relationships between features, may suffer from overfitting due to reliance on specific models and training processes and typically entails significant computational overhead. Embedded feature selection, while adapting well to model structures and considering interactions among features, may increase the risk of overfitting, sacrificing interpretability, and escalating computational complexity due to its high dependency on specific models. Thus, it presents challenges in balancing feature relationship comprehension and generalization ability. Although hybrid feature selection methods may mitigate the drawbacks of various feature selection methods to some extent, most existing methods, when handling hypertension gene expression data, assume independence among genes, neglecting the intricate regulatory network relationships among genes, which are crucial for effectively identifying biomarkers.Therefore, utilizing feature selection methods for hypertension genetic biomarker identification still entails certain

limitations.

Comment #4: Strengthen the literature review by including key publications on predicting marker genes associated with hypertension, such as the works by Gao et al. and Jiang et al., emphasizing their methodologies and findings.
1.Gao Y, Qi GX, Jia ZM, Sun YX. Prediction of marker genes associated with hypertension by bioinformatics analyses. Int J Mol Med. 2017 Jul;40(1):137-145. doi: 10.3892/ijmm.2017.3000. Epub 2017 May 25. PMID: 28560446; PMCID: PMC5466388.
2. Jiang C, Jiang W. Lasso algorithm and support vector machine strategy to screen pulmonary arterial hypertension gene diagnostic markers. Scott Med J. 2023 Feb;68(1):21-31. doi: 10.1177/00369330221132158. Epub 2022 Oct 17. PMID: 36253715.
Response #4： Thank you for the reviewers' suggestions. We have included relevant literature studies in the introduction, emphasizing their methodologies and findings. Please refer to the first response for details.

Comment #5: In the introduction, elaborate on the proposed approach's foundation in deep graph clustering. Discuss various clustering methods and their pros and cons, including Deepwalk, Node2vec, SDNE, and Weighted Correlation Network Analysis (WGCNA).
Response #5： Thank you for the reviewers' suggestions. In the introduction section, we have added the advantages and disadvantages of Deepwalk, Node2vec, SDNE, and Weighted Gene Co-expression Network Analysis (WGCNA) methods. The specific modifications are as follows:

...A popular method for this purpose is Weighted Gene Co-expression Network Analysis (WGCNA), which introduces the concept of soft threshold and realizes the construction of the network based on the expression similarity between genes[4]. It offers unique advantages in dealing with multi-sample biomarker screening. However, this method ignores potential local expression relationships that may exist only among certain genes and inadequately handles higher-order associations (such as nonlinear relationships or multivariate interactions), potentially missing some deeper connections within complex biological systems....

... Currently, researchers have proposed many methods of graph representation learning, such as DeepWalk[10] and Node2vec[11], which capture similarities between nodes based on random walks and word embedding techniques, and SDNE[12], which utilizes neural networks to learn low-dimensional representations of nodes while preserving the network's topological structure. Although these methods demonstrate powerful capabilities in capturing local neighborhood structures of nodes and learning implicit relationships between nodes and can effectively improve the performance and efficiency of subsequent biomarker identification tasks, they still lack complete modeling of the global topological structure of gene graph networks and ignore node attribute information and edge weight information, thereby

limiting their potential in comprehensively understanding and mining the deep structure-function relationships of gene expression networks...

Comment #6: Explain the criteria used for selecting the highest correlations among all pairs of genes. Specify the threshold considered as "highest" and clarify the process of creating the graph network by merging relevant edges (eGp and eGs).

Response #6: Thank you for the reviewers' suggestions. In Section 2.2, we improved the pruning threshold for networks constructed based on gene similarity and clarified the network creation process through pseudocode. The specific modifications are as follows:

... Specifically, we removed edges with weights below a certain threshold. For the selection of this threshold, we set it to a value obtained in the absence of isolated points. The resulting pruned graph network is denoted as e^{G_p}. Meanwhile, we utilized the physical interaction information from the STRING database as prior knowledge of gene interactions. Specifically, genes were imported into the STRING plugin database embedded within the Cytoscape software to obtain a network of gene interactions (with interaction scores greater than 0.4), denoted as e^{G_s}. Then, the e^{G_p} and e^{G_s} were merged, and duplicate edges were removed to form the final graph network, denoted as G. The specific process is detailed in Algorithm 1...

Algorithm 1: Construction of Network G

Input: df_1, df_2; //Data frames that represent the graphs e^{G_p} and e^{G_s}, respectively, composed of columns 'fromNode', 'toNode', and 'weight'.
gene_names. //list of genes under investigation.

Output: G

```
df2_filtered = []
for each row in df_2:
    if row['fromNode'] in gene_names and row['toNode'] in gene_names then:
        add row to df2_filtered;
    end if
end for
merged_df ← df_1    //copy the entire contents of df_1 into merged_df
for each row2 in df2_filtered:
    is_common_edge ← False
    for each row1 in merged_df:
        if row1['fromNode'] == row2['fromNode'] and row1['toNode'] == row2['toNode'] then:
            row1['weight'] += row2['weight'] //If edges exist in merged_df, the weights are updated
            is_common_edge←True
            break
        end if
    end for
    if not is_common_edge then:
        add row2 to merged_df    //If the edge does not exist in merged_df, it is added
    end if
end for
```

```
21:merged_df['weight'] /= 2.0;
22: G←merged_df;
23: return G.
```

Comment #7: Enhance clarity in the methodology by providing a detailed pseudocode, step by step, color each stage. Encourage sharing the code and dataset on GitHub for transparency.
Response #7： Thanks for this comment from the reviewer. As per the reviewers' recommendations, detailed pseudocode has been added to enhance the clarity of the methodology, including the process of merging graph networks and module partitioning. The dataset and partial code have also been shared on GitHub (https://github.com/lzj2051303710/Hy_DeepGCFS). Upon manuscript acceptance, the entire codebase will be made available for sharing.

Comment #8: Explain why Mutual Information Feature Selection (MIFS) is used in the final step, providing justification for choosing it over other feature ranking methods.
Response #8： Thank you for the reviewers' suggestions. Due to its effectiveness in quantifying the strength of associations between features, mutual information feature selection is particularly adept at revealing nonlinear relationships between gene expression and phenotype changes. Studies of complex biological systems, gene functions, and interactions often exhibit high levels of complexity and uncertainty. Mutual information feature selection provides a novel, statistically dependent perspective for identifying feature genes. Compared to machine learning model-driven feature selection methods such as random forests, SVM, and decision trees, mutual information can more comprehensively reflect the global correlation between features and target variables without establishing complex predictive models. Furthermore, it does not require parameter settings, reducing the subjective impact on results. Therefore, we employed mutual information feature selection (MIFS) in the final step.

We have added the rationale for selecting MIFS in Section 2.7. The specific modifications are as follows:

... Finally, considering that the MIFS method can effectively reveal the complex nonlinear relationship between gene expression and phenotypic changes, it does not require predefined parameters, thus reducing the potential impact of human factors on the results. Therefore, we selected MIFS to further optimize the genes involved in the feature subset. We selected the top 10 genes as potential biomarkers based on this refinement...

Comment #9: Compare the proposed approach's performance with at least two gene prioritization methods, considering AUC and Gene Ontology (GO) analysis.
Response #9： Thank you for the reviewers' suggestions. We have conducted a performance comparison analysis of our proposed method with four other methods. The experimental results are presented in Table 5.

Table 5 Comparison with published methods

literature	Method	AUC
Gao[3]	DEG+cytoHCA	90.75%
Li[5]	WGCNA	92.50%
Jiang[9]	DEG+SVM+Lass0	95.00%
Errington[30]	HybridFS	87.50%
DeepGCFS	DeepGCFS	97.50%

Comment #10: In GO and KEGG pathway analyses, please perform separate assessments for up-regulated and down-regulated genes.

Response #10： Thank you for the reviewers' suggestions. As this study primarily focuses on analyzing the overall functional characteristics of gene modules after clustering rather than based on directional changes in gene expression, it does not involve upregulated or downregulated genes. The purpose of the GO and KEGG pathway analyses is to examine the functionality of each gene module.

Comment #11: Utilize commonly used cross-validation (5 or 10 folds) instead of 3. Draw a heatmap of identified biomarkers and provide details on the number of genes and edges in the first step.

Response #11： Thank you for the reviewers' suggestions. Based on the sample size, we adopted the Leave-one-out cross-validation. In section 3.6, we have provided detailed information regarding gene and edge counts. Additionally, a heatmap depicting identified biomarkers has been added to section 3.7, as shown in Fig 5.

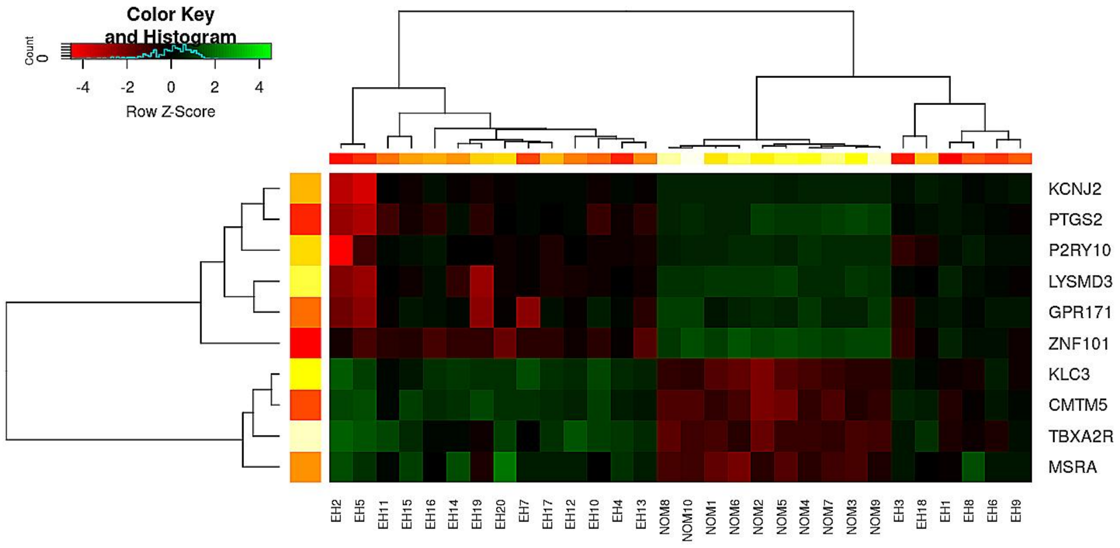

Fig5 Heat map analysis of the bimarkers by the proposed method

Comment #12: Compare the performance of the proposed approach with WGCNA.

Response #12: Thank you for the reviewers' suggestions. We have compared the performance of our proposed method with WGCNA, as shown in Section 4.

Comment #13: For visualizing gene modules, please utilize the widely-used STRING platform, incorporating color-coding to distinguish between up-regulated and down-regulated genes.
Response #13： Thank you for the reviewers' suggestions. In Section 3.6, we utilized the STRING platform to visualize gene modules and employed color coding to distinguish upregulated and downregulated genes.

Special thanks to you for your good comments！

SUGGESTIONS FROM REVIEWERS

REVIEWER #2

Comment #1: Dataset:The dataset used for this problem - a graph-theoretic machine learning analysis to identify biomarkers of hypertension - seems to be rather small - just a handful of HT and normal samples. A larger dataset may be necessary; please see comment on benchmarking.
Response #1: Thank you for the reviewers' suggestions. Due to the limited availability of transcriptomic data on hypertension submitted to both the SAR and GEO databases and the small sample sizes within each dataset, our study faces a scarcity of samples. To address the instability issues arising from small sample sizes during network construction, we have augmented our graph network construction by incorporating prior knowledge from the STRING database. Hypertension is a multifactorial disease with complex etiology. Despite extensive experimental and theoretical research conducted by scholars on its pathogenesis and treatment, the underlying mechanisms of hypertension remain unclear. To elucidate the molecular mechanisms of hypertension, it is essential to discover new biomarkers associated with hypertension. Our analysis identified ten potential gene biomarkers, achieving a favorable classification performance with an AUC of 97.50%. Validation using the GSE113439 dataset as an external dataset confirmed the robust classification performance when utilizing the ten gene biomarkers as features, with an AUC value of 95.45%. Therefore, our analysis holds significance for further understanding the molecular mechanisms of hypertension.

In recent years, many researchers have studied disease expression data in the GEO database to understand the pathogenesis of diseases. For example, Yang. et al. downloaded the GSE53987 dataset from the GEO database, studied 36 samples, and reported their results with an article titled‘Identification of Hub Genes and Key Pathways Associated With Bipolar Disorder Based on Weighted Gene Co-expression Network Analysis Front. Physiol. in 2019; Zhao. et al. downloaded two datasets, GSE12021 and GSE55235, from the GEO database and integrated them into 39

samples for research and screening for some pivot genes related to osteoarthritis. An article titled ‘Integration of Gene Expression Profile Data to Screen and Verify Hub Genes Involved in Osteoarthritis’ reported the result in Biomed. Res. Int. in 2018. Thus, our study remains relevant and meaningful.

Comment #2: Validation:The authors have used a GEO dataset for validation, but have performed three-fold cross-validation with the biomarkers. This is not valid. Even the biomarkers do not replicate on the validation dataset.
There is no train-test split either, and the authors may consult with a statistician for proper validation of their models.
Response #2： Thank you for the reviewers' suggestions. Based on the sample size and recommendations from statisticians, we adopted the Leave-one-out cross-validation.

Comment #3: Benchmarking with published models: Many models exist for prediction of hypertension. The authors have not compared their results with any of the existing models. Existing methods might perform better, this may be studied.
Response #3： Thank you for the reviewers' suggestions. We have conducted a performance comparison analysis of our proposed method with other methods in the literature. The experimental results are presented in the table.

Comment #4: Reproducibility:No stepwise results have been shared, and data supporting the conclusions has not been shared. No models have been shared, and work cannot be reproduced at all. Even the authors' biomarker replication efforts do not succeed with the validation dataset.
Response #4： Thank you for the suggestions from the reviewers. To enhance the clarity of the methodology, we have included detailed pseudocode. Additionally, we have shared the dataset and partial code on GitHub (https://github.com/lzj2051303710/Hy_DeepGCFS). Upon acceptance of the manuscript, we will make the entire codebase available for sharing.

Special thanks to you for your good comments！
