# Supplementary material for: Identification of hypertension gene expression biomarkers based on the DeepGCFS algorithm
Source: PLoS One. 2025 Jan 24;20(1):e0314319. doi: 10.1371/journal.pone.0314319 (PMC11761172; doi:10.1371/journal.pone.0314319)
Supplement: Attachment — Submitted filename: Response to Reviewers.docx [file pone.0314319.s007.docx]

**Response Letter**

(Manuscript ID: PONE-D-23-39381R1)

**Dear Hui Li and Reviewers,**

Thank you very much for taking the time to review our manuscript (Title: Identification of Hypertension Gene Expression Biomarkers Based on the DeepGCFS Algorithm [PONE-D-23-39381R1]). We sincerely appreciate all your insightful comments and suggestions. In this revision, we have addressed all the points raised. We hope that the revised manuscript now meets the publication standards of ***PLOS ONE***. The main corrections in the paper and our responses to the reviewer’s comments are as follows:

**REVIEWER #2**

**Comment #1:**LOOCV remains a form of cross-validation, which is unsuited for external validation. The model already built need not be cross-validated. How does that make sense?
**Response #1:** Thank you very much for your valuable comments and for taking the time to review our revised manuscript. We understand your concerns regarding the use of LOOCV and its limitations for external validation. To clarify, our external dataset was not used to validate the model itself but rather to assess the identified gene biomarkers. Specifically, our aim was to confirm that the biomarkers identified in our study are also hub genes in other independent datasets. Due to the relatively small size of the external dataset, we employed the leave-one-out cross-validation (LOOCV) method to evaluate the classification performance of the identified biomarkers. LOOCV enabled us to fully utilize the available data and ensure the reliability of the classification results, even with limited samples. That is, this process was focused on validating the consistency of the identified biomarkers, not the model. We hope this clarifies our approach and addresses your concerns. Thank you again for your thoughtful feedback.

**Comment #2:**The authors may be referring to Table 5. From Table 5, it is clear that many previous methods had been developed for this problem. Benchmarking all these models requires a common dataset for reference and evaluation. It is is not clear from the presentation if all the models were evaluated on the same dataset. This may be asserted if true. If not, then it is not meaningful to present this comparison.

**Response #2:** Thank you for your valuable comment. We agree that it is essential for a fair comparison to benchmark all models on the same dataset. In response to your concern, we would like to clarify that all the models presented in Table 5 were indeed evaluated on the same dataset. To improve the clarity of this point, we have revised the manuscript to explicitly state this fact in the relevant section. The following sentence has been added to the revised manuscript: “All the models listed in Table 5 have been benchmarked on the same dataset, ensuring consistency and fairness in the comparison” (in 3.8 Algorithm Performance Comparison). We hope this revision addresses your concern and clarifies the methodology used for the model evaluation in our study.

**Special thanks to you for your good comments！**

**REVIEWER #3**

**Comment**: In the revised version, my former comments have been addressed. I have no critical comments except that the wet-lab experiments for validating these findings. I think the authors will conduct them in the near future and they discuss this important issue in this paper.

**Response****:**

Thank you very much for taking the time to review our revised manuscript and for your positive feedback. We are pleased to hear that our revisions have addressed your concerns. We fully agree with your assessment regarding the importance of wet-lab experiments to validate our findings. As you correctly pointed out, these experiments are essential for confirming the biological relevance of our results. In future work, we plan to continue our research by incorporating experimental validation to further confirm the biological relevance of our findings. We sincerely appreciate your understanding and constructive feedback.

**Special thanks to you for your good comments！**

Thanks again!

Yours Sincerely,

Zongjin Li

E-mail: 202033341012@stu.qhnu.edu.cn
